# Houttuynia cordata thunb. alleviates inflammatory bowel disease by modulating intestinal microenvironment: a research review

**DOI:** 10.3389/fimmu.2023.1306375

**Published:** 2023-11-22

**Authors:** Si Wang, Lei Li, Yuhan Chen, Qian Liu, Shengyu Zhou, Ning Li, Yueying Wu, Jiali Yuan

**Affiliations:** ^1^ First Clinical School of Medicine, Yunnan University of Chinese Medicine, Kunming, Yunnan, China; ^2^ College of Basic Medical Sciences, Yunnan University of Chinese Medicine, Kunming, Yunnan, China; ^3^ Yunnan Provincial Key Laboratory of Integrated Traditional Chinese and Western Medicine for Chronic Disease in Prevention and Treatment, Yunnan University of Chinese Medicine, Kunming, Yunnan, China

**Keywords:** Houttuynia cordata thunb., homology of medicine and food, intestinal microenvironment, immunity, inflammatory bowel disease

## Abstract

Inflammatory bowel disease (IBD) is a complex group of chronic intestinal diseases, the cause of which has not yet been clarified, but it is widely believed that the disorder of the intestinal microenvironment and its related functional changes are key factors in the development of the disease. Houttuynia cordata thunb. is a traditional plant with abundant resources and long history of utilization in China, which has attracted widespread attention in recent years due to its potential in the treatment of IBD. However, its development and utilization are limited owing to the aristolochic acid alkaloids contained in it. Therefore, based on the relationship between the intestinal microenvironment and IBD, this article summarizes the potential mechanisms by which the main active ingredients of Houttuynia cordata thunb., such as volatile oils, polysaccharides, and flavonoids, and related traditional Chinese medicine preparations, such as Xiezhuo Jiedu Formula, alleviate IBD by regulating the intestinal microenvironment. At the same time, combined with current reports, the medicinal and edible safety of Houttuynia cordata thunb. is explained for providing ideas for further research and development of Houttuynia chordate thunb. in IBD disease, more treatment options for IBD patients, and more insights into the therapeutic potential of plants with homology of medicine and food in intestinal diseases, and even more diseases.

## 1 Introduction

Inflammatory bowel disease (IBD) is an autoimmune and gut-related inflammatory disease mainly encompassing Crohn’s disease (CD) and Ulcerative colitis (UC), with chronic relapsing and remitting characteristics, characterized by inflammatory damage to the intestinal mucosa as the main pathological feature (1). Surveys by Ng SC (2) and Porter RJ (3) indicate that IBD’s prevalence is predominantly in developed regions like North America and Europe, with incidence rates exceeding 0.3%. However, since 2015, areas represented by China and Brazil, have witnessed a rapid surge in IBD cases. This uptrend, fueled by accelerated industrialization and dietary shifts, has resulted in annual growth rates peaking at 14.9%. By 2030, global IBD patient numbers are projected to reach 20.25 million.

The etiology of IBD remains unclear. Current research suggests potential causes, such as disturbances in the intestinal microenvironment, genetics, and environmental factors, all contribute to the onset and progression of IBD (4). Disturbances in the intestinal microenvironment and associated functional changes are regarded as having the closest links with the pathogenesis, recurrence, and drug resistance of this refractory disease (5). Therefore, the primary therapeutic approach for IBD focuses on inducing clinical symptom remission (6). While treatments demonstrate efficacy, nearly 30% of patients require surgical intervention due to uncontrolled disease progression. Coupled with the historically insufficient attention to IBD’s disability rates, its prevention, treatment, and cure rates lag behind other digestive diseases, posing a significant burden to global health. Hence, there is an urgent need for more safe and effective drugs to provide novel therapeutic strategies for IBD (7).

Traditional Chinese medicine classifies IBD under the categories of “diarrhea” and “prolonged dysentery”, and believes that its main causes are the accumulation of pathological products such as phlegm and blood stasis in the intestines, which then lead to the formation of heat toxin and damage to the intestinal vein. Therefore, treatment should focus on promoting blood circulation, removing blood stasis, clearing heat, and eliminating carbuncles. When selecting medications, the “three principles of adaptation” theory of traditional Chinese medicine is often used as a guide. Therefore, based on regional characteristics, it has become an effective way to prevent and treat IBD to find more suitable Chinese herbal medicines for human body constitution from natural plants that are abundant in local growth and have a long history of safe consumption, which can be used to treat IBD.

Houttuynia cordata thunb. (HC), the fresh or dried above-ground part of the plant Jicai from the Saururaceae family, primarily grows and eats in southwest China, including Sichuan, Yunnan, and Guizhou provinces (8). This dual-purpose medicinal and edible plant is recognized for its properties of clearing heat, treating diarrhea, reducing swelling, healing sores (9). According to the theory of meridian tropism, which posits that certain drugs selectively act on specific organs (10), HC mainly targets the lung meridian, emphasizing the modulation of lung function. However, in traditional Chinese medicine theory, the lungs and the large intestine share an intimate “Exterior-interior correlation” relationship (11), coupled with recent advancements in modern medicine studying the “lung-gut axis”, there has been a growing interest in utilizing HC and its active components for IBD treatment, with a focus on rectifying the imbalanced intestinal microenvironment to alleviate IBD. The clinical complete remission rate can reach 95.2%, which is superior to conventional drug intervention groups such as sulfasalazine (12).

## 2 The relationship between intestinal microbial environment and IBD

The intestinal microenvironment, constituted by a substantial number of microbial populations in specific proportions, plays a crucial role in protecting intestinal mucosa, absorbing and transmitting energy, and metabolizing nutrients. Alterations in this microenvironment can induce pathological reactions in the intestine (13). Recent advancements in research have confirmed that the ecological imbalance in IBD, resulting from changes in the composition and functionality of intestinal microbes, could be the pivotal trigger in the pathogenesis of IBD (14).

### 2.1 Relationship between intestinal flora and IBD

The gut microbiota represents a dynamic interplay determined collaboratively by the host, microbial self-selection, and environmental factors. This organic consortium is particularly crucial in host immune defense, metabolic nutrient absorption, and the processes of health maintenance and disease alleviation. It has been termed the “forgotten organ” (15) and the “eighth human organ” (16).

The human digestive tract harbors over a trillion commensal microbes, reaching concentrations of 10^11^ to 10^12^ cells per gram of luminal content, with over 99% predominantly belonging to the phyla *Firmicutes*, *Bacteroidetes*, *Actinobacteria*, and *Proteobacteria* (17, 18). The mutual interactions among these bacteria, characterized by resilience and adaptability, maintain a beneficial dynamic equilibrium in the intestinal microenvironment. Simultaneously, they promote the differentiation and maturation of intestinal epithelial cells and immune cells (18), providing a barrier against pathogenic infections, ultimately safeguarding host health (19).

**Figure 1:**
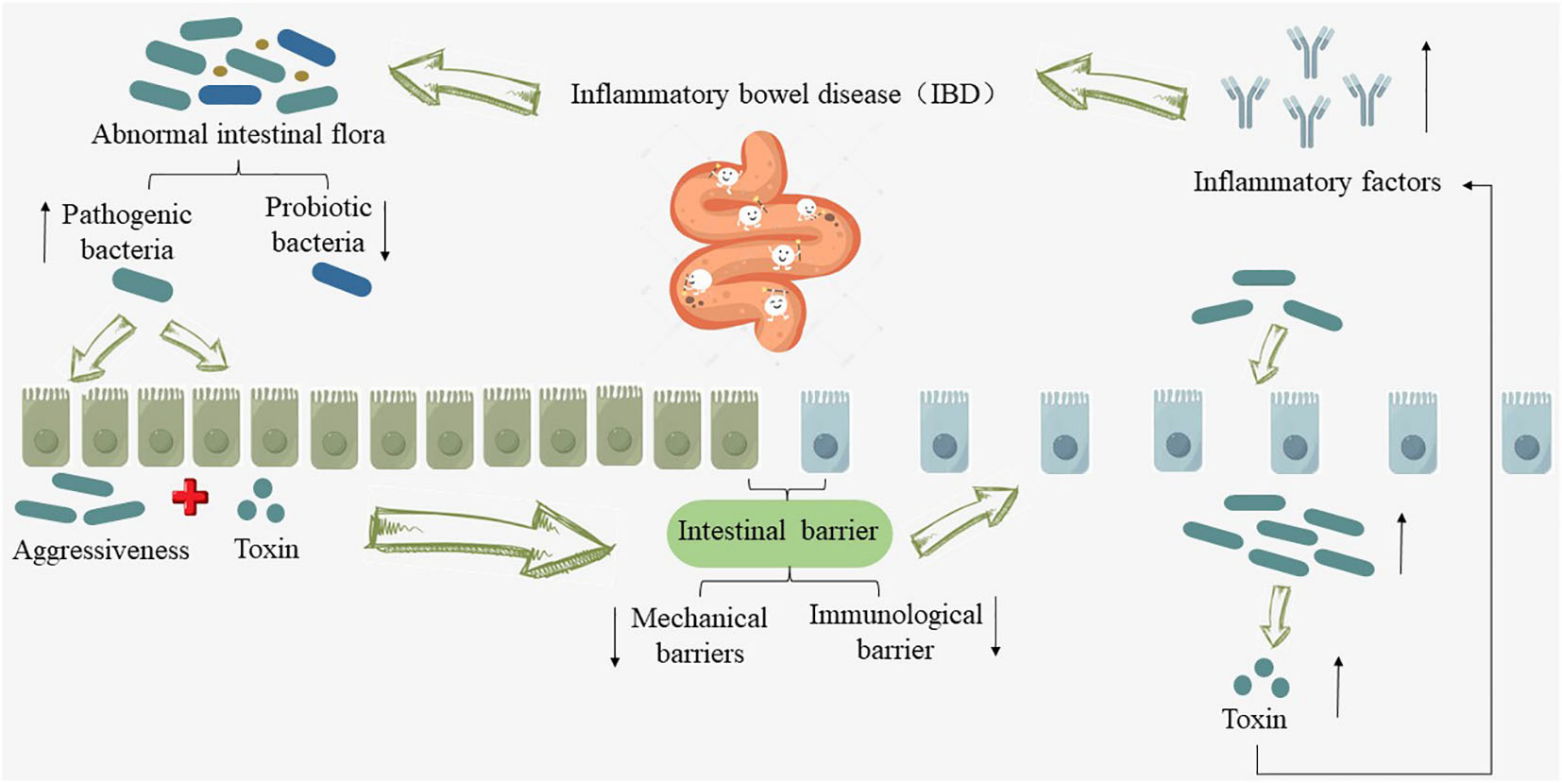
Relationship between intestinal flora and IBD. “↑” indicates an increase, “↓” indicates a decrease.

However, when IBD develops, the normal structure and abundance of the intestinal microbiota are disrupted. The quantity of pathogenic bacteria, exemplified by adherent-invasive *Escherichia coli*, increases (20), while beneficial probiotics such as *Bifidobacteria* and *Lactobacilli* decrease (21). The mechanical barrier of the intestinal mucosa and the immune barrier are compromised during the invasion and toxin secretion by pathogenic bacteria, consequently increasing intestinal mucosal permeability, creating conditions for microbial translocation (20). The transferred bacteria and their released endotoxins further induce the expression of additional inflammatory factors, disrupting the normal immune balance in the gut and activating abnormal immune responses within the host (22), which exacerbates intestinal inflammation and mucosal tissue damage in IBD, promoting the progression and prolongation of the condition (as shown in **Figure 1**).

Herrera-deGuise C et al. (23) conducted a study involving a total of 184 volunteers who provided 111 fecal samples. These samples were collected from individuals in various health conditions, including long-term remission patients, short-term remission patients, patients in acute phase, and healthy control subjects. Following a microbiome analysis, the researchers discovered that compared with healthy control subjects, the intestinal flora abundance and diversity in IBD patients were low, while the intestinal microflora in long-term remission IBD patients had substantial recovery in abundance and diversity, and approached that of healthy individuals. However, when IBD patients underwent fecal microbiota transplantation from healthy donors, their clinical symptoms, such as diarrhea, abdominal pain, rectal bleeding, as well as intestinal mucosal inflammation and Mayo scores, significantly improved compared to the placebo group (24). Furthermore, through metagenomic techniques aimed at identifying differential microbial communities, studies by Javed NH et al. (25) and Zhang B et al. (26) have also confirmed that direct supplementation of probiotics such as *Bifidobacteria* can also protect the structure of the intestinal mucosal goblet cells and epithelial cell layers, thereby reducing the structural damage of the mucosal barrier, regulating the expression of immune-related cells such as Th17 cells, and thus alleviating the symptoms of IBD. These studies collectively demonstrate the close interplay between the gut microbiota and the development of IBD. Therefore, modulating the abundance and diversity of the gut microbiota to restore the dynamic balance between the gut microbiome and the host immune defense mechanisms is a critical therapeutic approach in the clinical search for methods to inhibit or slow down the occurrence and persistence of IBD (27).

### 2.2 Relationship between intestinal flora metabolites and IBD

The metabolites of intestinal flora originate from the small molecules produced by the intermediate or final products of intestinal flora metabolism, and are present in serum, urine, feces, and other biological tissues, exerting profound effects on the immune maturation, metabolic homeostasis, and maintenance of intestinal mucosal integrity of the body (28).

With the continuous development of genomics and metabolomics technologies, it has been observed that various gut microbiota metabolic products in individuals with IBD undergo significant alterations when compared to healthy individuals (29). These alterations can amount to over 2700 different metabolites (30). Specifically, disruptions in bile acids (BA), short-chain fatty acids (SCFAs), and tryptophan metabolism have been demonstrated to be closely associated with the occurrence and development of IBD (31). They play a role in the differentiation and functional regulation of immune cells involved in IBD development, such as Treg, Th17, CD4 cells, CD8 cells, B cells, and others (32).

#### 2.2.1 Relationship between BA metabolism disorder and IBD

BA are amphipathic molecules with hydrophobic (β-side) and hydrophilic (α-side) surfaces. They exhibit detergent properties, generated in the liver through cholesterol metabolism and ultimately metabolized by the intestinal microbiota (33). They consist of primary bile acids (PBAs) and secondary bile acids (SBAs), participating in the host’s enterohepatic circulation and microbial metabolic pathways. They facilitate the solubilization of lipids in micelles, promoting the emulsification and absorption of dietary fatty acids and cholesterol (34). Additionally, they regulate intestinal epithelial cell proliferation, intestinal mucosal barrier function, thus contributing to host immune modulation (35).

**Figure 2:**
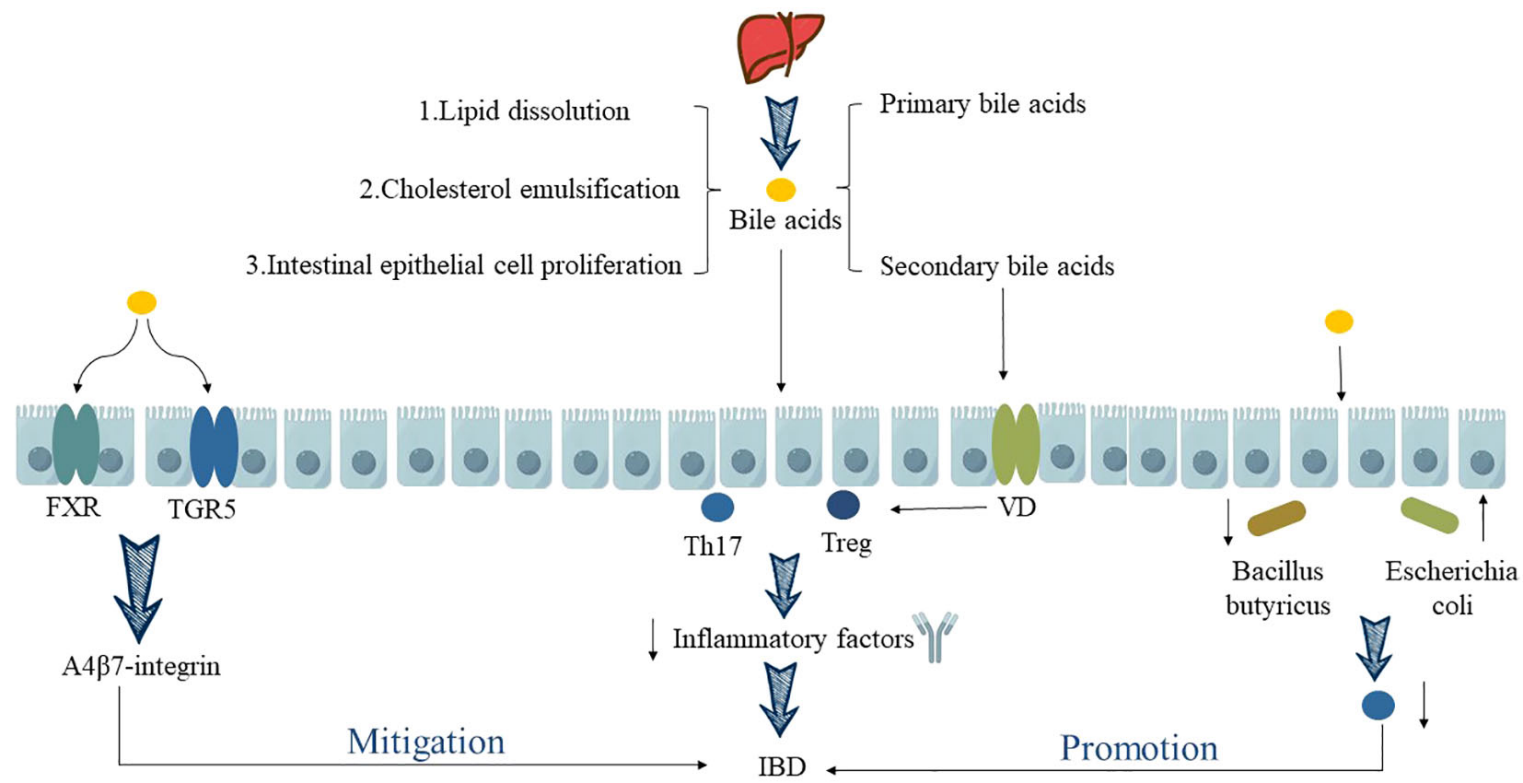
Relationship between BA metabolism disorder and IBD. “↑” indicates an increase, “↓” indicates a decrease.

Over the past decade, the pivotal role of gut microbiota metabolites has been highlighted and investigated in various immune-mediated diseases, particularly in the case of IBD. Long XQ et al. (34) discovered that during the pathogenesis of IBD, BA can directly or indirectly influence the abundance and diversity of the gut microbiota, subsequently modulating the host immune response to ameliorate IBD symptoms or delay disease progression. BA, such as through the activation of FXR and TGR5 expression, impact the anti-α4β7-integrin response in IBD, thereby promoting the restoration of gut microbiota homeostasis (36). This regulation extends to the balance between Th17 and Treg cells (35), ultimately suppressing or alleviating intestinal inflammation in IBD. When there is dysregulation in BA metabolism, it significantly affects the differentiation and renewal of intestinal stem cells, leading to dysbiosis in the gut microbiota. This dysbiosis results in a marked reduction in the abundance of *Firmicutes*, *Clostridia*, and an increase in pathogenic bacteria like *Escherichia coli*, *Enterococcus*, *Klebsiella*, and *Streptococcus* (37). This disruption severely impairs the function of the intestinal mucosal barrier. Additionally, it leads to decreased expression of Th17 cell-related genes, ultimately emerging as a decisive factor in the malignant progression of IBD (35). This has also been confirmed by research by Kubota H et al. (38), who showed that after oral administration of lithocholic acid, a secondary metabolite of intestinal flora, the body weight and disease activity index (DAI) of Dextran Sulfate Sodium (DSS)-induced experimental colitis mice were significantly improved, and the pathological manifestations of colon tissue, such as infiltration of inflammatory cells and loss of goblet cells, were alleviated. The mechanism is mainly that lithocholic acid can participate in maintaining the homeostasis of Treg cells by activating vitamin D receptors in immune cells, thereby inhibiting intestinal inflammatory reactions and alleviating UC (as shown in **Figure 2**).

#### 2.2.2 Relationship between SCFAs metabolism disorder and IBD

SCFAs are a class of fatty acids with fewer than six carbon atoms produced through the fermentation of carbohydrates in the proximal colon and protein hydrolysis in the distal colon by various anaerobic bacteria in the gut. The major components of SCFAs include acetic acid, propanoic acid and butyric acid (39). Among these, butyric acid is primarily produced by *Firmicutes*, while acetic acid and propanoic acid are generated by *Bacteroidetes* (40). Research has demonstrated that SCFAs, as the predominant metabolic products of the gut microbiota, can fulfill 60%-70% of the energy requirements of colonocytes. Furthermore, butyric acid, one of the SCFAs, is involved in numerous signaling pathways in intestinal immune cells and epithelial cells. It can regulate the host’s immune homeostasis and enhance intestinal mucosal barrier function. Therefore, butyrate is emerging as a focal point in potential therapeutic strategies for IBD (41).

**Figure 3:**
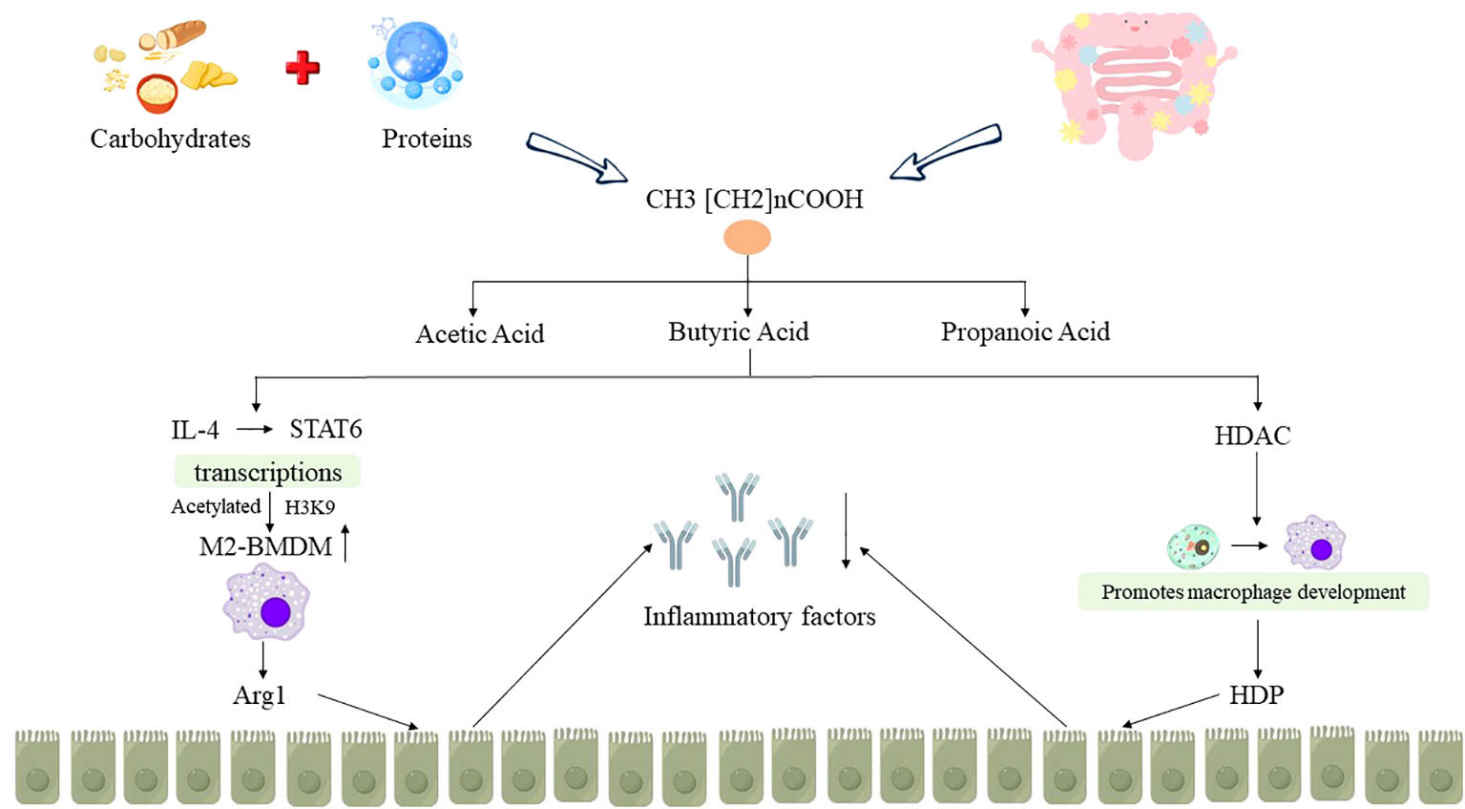
Relationship between SCFAs metabolism disorder and IBD. “↑” indicates an increase, “↓” indicates a decrease.

Macrophages are crucial components of the innate immune system and can be categorized into two main types, M1 and M2 macrophages. They play a pivotal role in shaping the inflammatory milieu in various disease contexts (42). M1 macrophages, induced by factors such as LPS, generate a plethora of pro-inflammatory cytokines which exacerbates tissue inflammation. On the other hand, M2 macrophages, induced by cytokines like IL-4 and IL-13, are characterized by the expression of Arginase-1 (Arg1) and play essential roles in mitigating tissue inflammation, promoting wound healing, and facilitating tissue repair (43). Ji J et al. (44) conducted animal experiments and found that butyric acid intervention in DSS-induced UC mice models enhances the polarization of M2-BMDM macrophages through the augmentation of IL-4-mediated STAT6 transcription and H3K9 acetylation. This process promotes Arg1 synthesis, facilitating the repair of damaged intestinal mucosal barriers and significantly reducing colonic inflammation in mice. In addition, butyrate has also been shown to promote the development of monocytes into macrophages by inhibiting histone deacetylase (HDAC), stimulate macrophages to increase the synthesis of endogenous host defense peptides (HDP), and affect intestinal immunity by maintaining the integrity of the intestinal mucosal barrier, providing epithelial energy supply, and reducing inflammatory expression, thereby alleviating IBD (45) (as shown in **Figure 3**).

#### 2.2.3 The relationship between dysregulation of tryptophan metabolism and IBD

Tryptophan is an essential amino acid for humans, which can be supplemented through the intake of lean meat, fish, dairy products, nuts, and seeds. It is produced in the gastrointestinal tract through three main pathways, namely the kynurenine pathway, the 5-hydroxytryptamine pathway, and the indole pathway, which play separate but complementary roles in regulating gastrointestinal function and influencing intestinal homeostasis, thereby interfering with the progression of IBD (46). Notably, research on the indole pathway in IBD has yielded more in-depth insights. It has been observed that the gut microbiota can convert 4% to 6% of intracellular tryptophan into indole and related indolic compounds, which serve as ligands for the aryl hydrocarbon receptor (AhR) and pregnane X receptor (PXR). This ligand-receptor interaction is instrumental in modulating mucosal immunity within the intestinal tract, maintaining intestinal environmental stability, and alleviating the progression of IBD (47).

Yang C (48) and Bischoff SC (49) discovered significant differences in tryptophan metabolism between UC patients and healthy volunteers through a comparative analysis. They found that UC patients had notably lower levels of tryptophan than healthy volunteers. Furthermore, in active UC patients, serum tryptophan levels were negatively correlated with the inflammatory markers ESR and CRP (*P* < 0.05). A large cohort study involving 535 IBD patients also yielded similar results. This study indicated that the severity of IBD disease activity was inversely related to tryptophan levels (50). Consequently, serum tryptophan levels hold monitoring value in the course of IBD and are of significant importance in IBD research.

To further investigate the mechanisms underlying tryptophan metabolism in IBD, Lamas B et al. (51) conducted relevant experiments and discovered that the potential mechanisms by which tryptophan metabolism ameliorates IBD primarily involve the AhR signaling pathway, the PXR signaling pathway, and the modulation of cytokines.

Firstly, the microbiota-AhR axis has been confirmed as a fundamental element in maintaining intestinal immune homeostasis. Tryptophan metabolites, such as indole-3-acetic acid, are potent bioactive substances influencing adaptive and innate immune responses. Their role in the body’s immune system mainly relies on the activation of the AhR signaling pathway, which is widely present in immune cells and intestinal epithelial cells and plays a regulatory role in intestinal mucosal immunity (52, 53). Therefore, after knockout of the IBD risk allele CARD9, it was observed that the metabolism of tryptophan decreased in mice, and it was difficult to catalyze tryptophan into AhR ligands, resulting in reduced release of IL-22 factors, making mice more susceptible to DSS-induced IBD. However, after inoculating mice with three strains of lactic acid bacteria that contain tryptophan or by intervening with AhR agonists, the intestinal inflammation in mice decreased, and IBD was alleviated (51).

**Figure 4:**
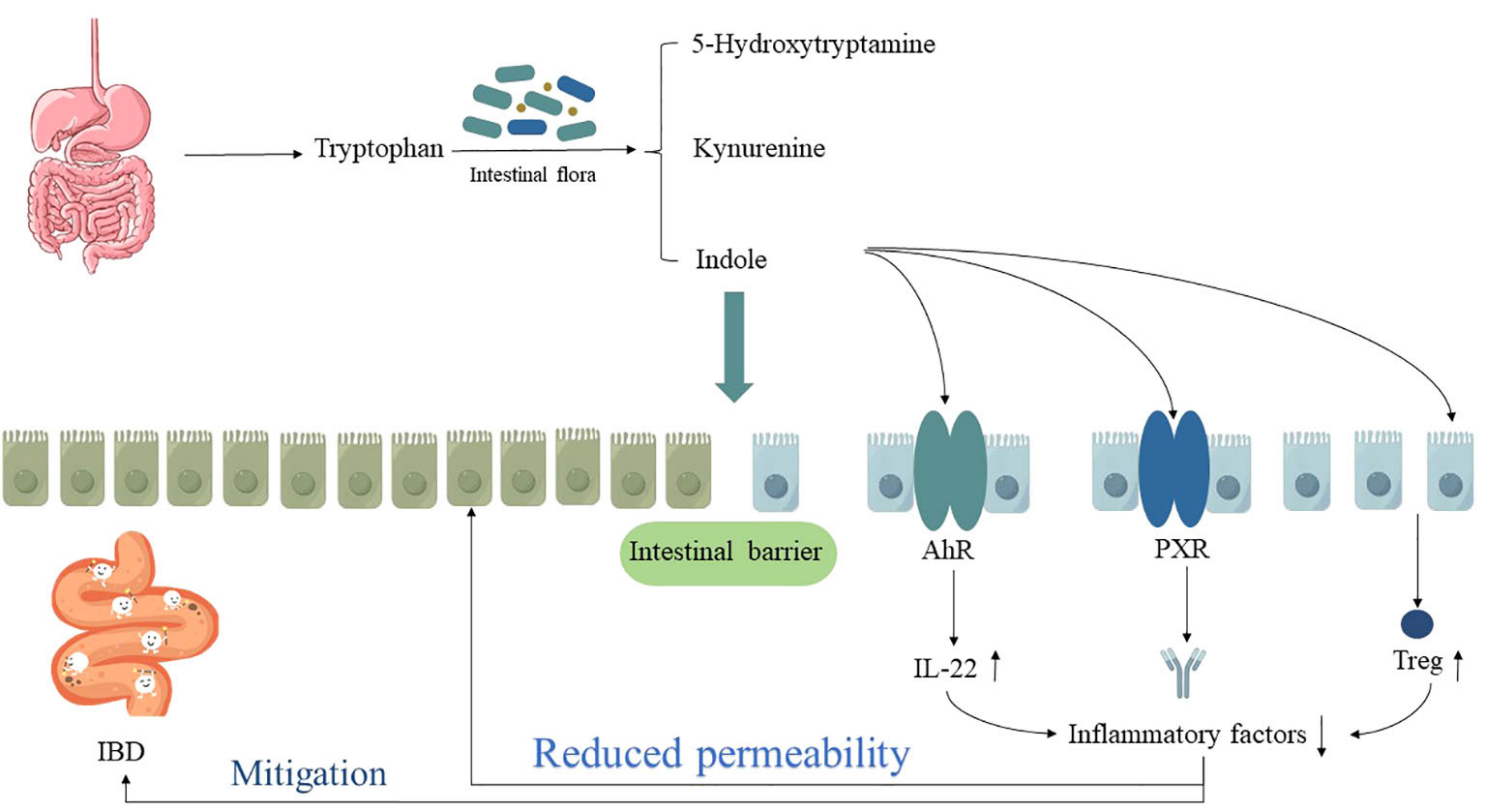
The relationship between dysregulation of tryptophan metabolism and IBD. “↑” indicates an increase, “↓” indicates a decrease.

Furthermore, the PXR signaling pathway has also been confirmed as a significant potential mechanism affecting the progression of IBD through its impact on tryptophan metabolism. PXR plays a role in regulating intestinal mucosal barrier function under homeostatic conditions and serves as a critical modulator of innate immune responses in the intestine and their responses to damage. In the absence of PXR, UC mice exhibit evident intestinal mucosal barrier “leakiness”, whereas mice without PXR defects can activate the PXR signaling pathway through tryptophan metabolite indole-3-propionic acid, resulting in reduced intestinal permeability and suppressed inflammatory responses, thereby alleviating the disease (54). Additionally, Ding X et al. (55) discovered that patients with IBD exhibit disruptions in tryptophan metabolism during the pathological process. However, by modulating tryptophan metabolism, it is possible to induce the differentiation of Treg cells through intestinal microbiota breakdown and the kynurenine pathway. This process, involving cell-cell contact and cytokine release, helps maintain the intestinal microenvironment homeostasis in IBD patients and alleviate inflammation damage to intestinal tissues (as shown in **Figure 4**).

However, while it has been confirmed that tryptophan metabolism is involved in the pathology and physiological processes of IBD, current research predominantly focuses on exploring the potential of tryptophan as a biomarker and its role in regulating immune and inflammatory responses (56). The specific mechanisms of tryptophan metabolism in the pathogenesis of IBD remain unclear. Additionally, further investigation is needed in the kynurenine pathway and the 5-hydroxytryptamine pathway.

## 3 The potential mechanism of HC in alleviating IBD through regulating the intestinal microenvironment

HC, a perennial herbaceous plant commonly associated with root nodules bacteria, derives its name from its distinctive fish-like odor permeating the entire plant. Typically thriving in wetlands, stream margins, and meadows (57), this plant usually exhibits a height ranging from 20 to 50 cm. Its leaves, either green or red, are oval or heart-shaped, measuring between 4 to 8 cm in length and 3 to 6 cm in width. The plant bears densely packed spicate flowers, with its slender rhizomes presenting nodes that are either glabrous or covered in short soft hairs (58).

HC is widely distributed in Asian regions, especially in Yunnan, Hunan, Hubei, Sichuan, and Guizhou provinces of China. It is primarily used as a food ingredient due to its richness in vitamins and minerals (59). With the enrichment of pharmacological research methods in recent years, the preparations containing HC, as well as the main chemical components and medicinal effects of HC, have been continuously explored. Among them, essential oils, flavonoids, alkaloids, and polysaccharides are the main chemical components with pharmacological activity in HC, and have been confirmed to have immune regulatory activity, anti-inflammatory, antiviral, antioxidant, and tumor cell proliferation inhibitory effects (60). They have therapeutic effects in diseases such as liver cancer (61), acute liver injury (62), avascular necrosis of the femoral head (61), asthma (63), pneumonia (64), alzheimer’s disease (65), and heart failure (66). Consequently, the plant has been recognized by the Chinese National Health Department as one of the most promising resources for both medicinal and culinary uses (67).

### 3.1 Essential oils

HC, often referred to as the “Chinese herbal antibiotic”, has been proven through research to possess anti-inflammatory, antioxidant, and antiviral properties. These effects have been attributed to its essential oils content. Furthermore, the essential oils constituents serve as the primary active components responsible for various pharmacological effects of the whole fish mint plant. These constituents include houttuynin, decanal, trans-caryophyllene, decanoic acid, camphene, β-pinene, lauraldehyde, α-pinene, limonene, nonanol, linalool bornyl acetate, methyl n-nonyl ketones, beta myrcene, monoterpene, 4-terpineol, caryophyllene oxide, phenylpropene derivatives, sesquiterpenes, and oxidized diterpenes (59).

*Candida albicans*, a common opportunistic pathogen found in the human intestinal tract, has been associated with the severity of UC. Cheng T et al. (68) found that the aldehyde and sodium bisulfite adduct of HC essential oils, sodium houttuyfonate (SH), can regulate the abundance and diversity of gut microbial flora by inhibiting harmful pathogens such as *Leiberella* and *Bacteroides*, and increasing beneficial bacteria, including bacteria producing SCFAs (*Lachnospiraceae_NK4A136_group*, *Intestinimonas*) and probiotics (*Lactobacillus* and *Alloprevotella*), thereby maintaining the stability of the intestinal microenvironment. It significantly improved the pathological signs of colon shortening and intestinal mucosal barrier injury in IBD mice infected with *Candida albicans*, significantly inhibiting the growth of *Candida albicans*. The mechanism of SH improving IBD was further confirmed in UC caused by *Salmonella typhimurium*, and was closely related to the inhibition of NF-κB signaling pathway (69) (as shown in **Table 1**).

**Table 1:** The potential mechanism of HC in alleviating IBD through regulating the intestinal microenvironment.

	Changes in intestinal micro-flora or metabolite levels		Therapeutic effect	References
	Promotes	Reduces		
Essential oils	*Lactobacillus* *Alloprevotella*	*Leiberella* *Bacteroides* SCFAs *Candida albicans* *Salmonella typhimurium*	Colon length↑ Colon tissue injury↓ MDA, MPO↓ TNF-α, IL-1β, IL-6↓ IL-10↑	(68, 69)
Polysaccharides	*Firmicutes* *Bacteroidetes*	*Proteobacteria*	Weight↑ colon length↑ TNF-α, IL-1β, IL-6↓ TLR4, NF-κB↓ Restore Th17 and Treg cell function	(70–72)
Flavonoids (Total flavonoids, Quercetin)	*Firmicutes*	*Bacteroidetes* *Proteobacteria*	Weight↑ Hematochezia↓ DAI index↓ colon length↑ Ulcerative surface↓ Inflammatory cell infiltration↓ The keratoid structure and mucosal wall thickness are clear IFN-γ, IL-1α, IP10, TNF-α 4↓ ALT, AST, CR, BUN↓	(73)
	*Bacteroides Bifidobacterium* *Lactobacillus* *Clostridia*	*Fusobacterium* *Enterococcus*	Hematochezia↓ IL-17, IL-6, TNF-α↓ Colonic histopathological score↓	(74)
Xiezhuo Jiedu Formula	*Lactobacillus* *Bifidobacterium*	*Enterobacteriaceae* *Enterococci*	Prevent recurrence of UC	(75, 76)

“↑” indicates an increase, “↓” indicates a decrease.

### 3.2 Polysaccharides

Plant polysaccharides are natural macromolecular compounds derived from plants. Studies have shown that plant polysaccharides not only promote the proliferation of beneficial bacteria in the gut, but also produce organic acids through fermentation in the gut, thereby reducing the pH of the gut and making it difficult for harmful bacteria in the gut to effectively utilize plant polysaccharides, thus exerting an inhibitory effect on the growth and reproduction of pathogenic bacteria (77), restoring the diversity of gut flora, regulating the structure of gut flora, and repairing the mucosal barrier to protect the gut (78).

With the increasing attention of researchers to dual-purpose natural plants for medicine and food, as well as the diversification of extraction methods, the polysaccharides in HC have been identified as crucial pharmacologically active components. These include rhamnose, galacturonic acid, galactose, and arabinose, which possess notable immunoregulation, intestinal protection, anti-inflammatory, and antioxidant activities, demonstrating increasing advantages in the treatment of refractory diseases (79, 80).

Cen L et al. (70) established an animal model of IBD using DSS induction and then intervened with HC polysaccharides. The results showed that HC polysaccharides can regulate intestinal flora, such as increasing the abundance of *Firmicutes* and *Bacteroidetes*, reducing the abundance of *Proteobacteria*, improving the intestinal microenvironment, and then intervening in the expression of inflammatory signaling pathways such as NF-κB, inhibiting macrophage infiltration (71), alleviating pathological damage to the colonic mucosal mechanical barrier, thereby regulating the function of Th17 and Treg cells, protecting colonic tissue, and significantly inhibiting general signs such as weight loss and shortening of the colon length in mice. After successfully replicating the UC model, Ping W et al. (72) administered intragastric intervention with HC polysaccharides solution to mice, and the results also confirmed that HC polysaccharides can increase the species richness of intestinal bacteria in UC mice, promote the recovery of intestinal flora abundance and structure, reduce the relative abundance of *Proteobacteria*, inhibit the expression of pro-inflammatory cytokines in the intestine, significantly increase the level of *Firmicutes*, restore the microecological homeostasis in the intestine, and repair the intestinal mucosal barrier, thereby improving UC (as shown in **Table 1**).

### 3.3 Flavonoids

Flavonoids in HC, similar to the essential oils found within the plant, have been proven to possess significant anti-inflammatory and antiviral activities, representing another major active ingredient underlined in relevant studies (81). These flavonoids include quercetin, rutin, hyperin, afzelin, quercitrin, isoquercitrin, kaempferol, quercetin hexoside, avicularin, apigenin, isorhamnetin, phloridzin, and quercetin-3-O-β-D-galactoside-7-O-β-D-glucoside (82).

Letian Y et al. (73) used a 3.5% DSS solution to continuously drink water to replicate the model of UC in mice, and then used cellulase-assisted ultrasonic extraction to extract total flavonoids from HC for intervention. The results showed that total flavonoids in HC could maintain the stability of intestinal microecology by regulating the proportion of dominant bacterial groups such as *Bacteroidetes*, *Firmicutes*, and *Proteobacteria* to varying degrees, thereby effectively improving the body weight of UC mice, reducing hematochezia, reducing DAI index, alleviating symptoms such as shortened colon, inhibiting the expression of serum inflammatory factors, and showing a dose-dependent effect (as shown in **Table 1**).

In addition, quercetin in HC, a flavonoid compound widely distributed in the plant kingdom, has also been shown to play a therapeutic role in the treatment of IBD. For example, Lin R et al. (74) showed that after quercetin intervention in a *Citrobacter rodentium*-induced IBD mice model, intestinal microbial diversity was reshaped, and the balance between bacterial populations was regulated. The number of *Bacteroides*, *Bifidobacterium*, *Lactobacillus*, and *Clostridia* increased, while *Fusobacterium* and *Enterococcus* decreased significantly, thereby promoting the restoration of local immune homeostasis in the intestine and significantly alleviating IBD symptoms such as hematochezia (as shown in **Table 1**).

### 3.4 HC preparation alleviates IBD by regulating the intestinal microenvironment

The Xiezhuo Jiedu Formula (XZJDF) is a herbal preparation based on HC used to treat UC (75). Siyu L et al. (76) found that after intragastric administration of the XZJDF in high, medium, and low doses to UC rats induced by a mixture of 2,4,6-trinitrobenzene sulfonic acid (TNBS) and 50% ethanol, the relative expression levels of *Lactobacillus* and *Bifidobacterium* in the high dose group were higher than those in the model group, while the relative expression levels of *Enterobacteriaceae* and *Enterococci* were lower than those in the model group. Furthermore, during the second modeling stimulation, the high dose group of the XZJDF showed a lasting effect on regulating the balance and stability of intestinal flora, further improving the defensive capacity of the intestinal mucosal barrier, and significantly improving the condition of UC rats. Therefore, it can be concluded that the HC preparation XZJDF can target intestinal flora, increase the relative content of *Lactobacillus* and *Bifidobacterium*, reduce the relative content of *Enterobacteriaceae* and *Enterococci*, make the beneficial bacteria dominate over the harmful bacteria, regulate the disorder of intestinal flora, and maintain the long-term stability of the intestinal microenvironment, thus maintaining the health of the body and delaying the recurrence of UC (as shown in **Table 1**).

**Figure 5:**
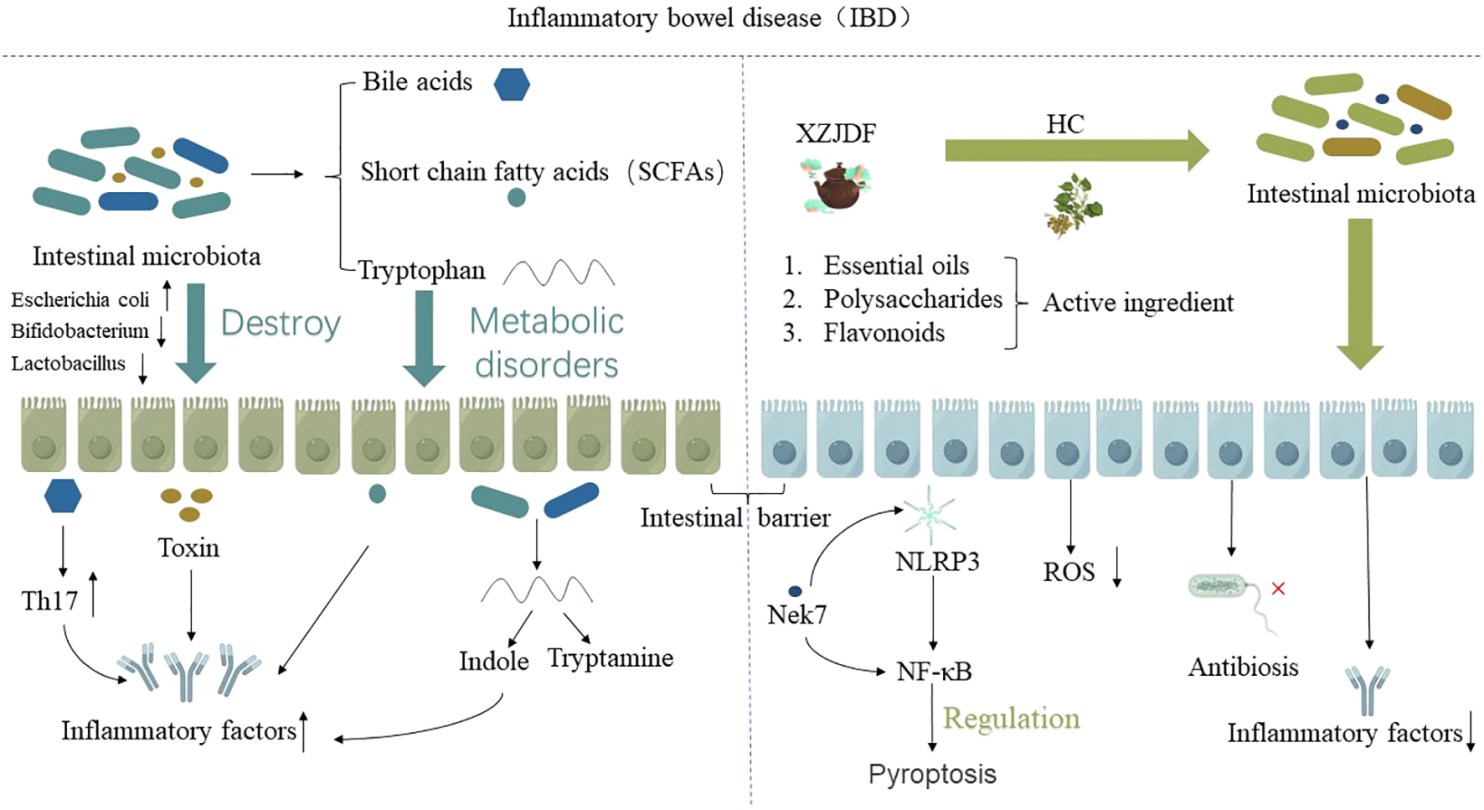
The potential mechanism of HC in alleviating IBD is mainly due to the active ingredients in HC, such as essential oils, polysaccharides, flavonoids, and HC-related preparations, which regulate the intestinal microenvironment, including regulating intestinal flora and its metabolites, thereby improving the body’s immunity to alleviate IBD.”↑” indicates an increase, “↓” indicates a decrease.

In summary, the potential mechanism of HC in alleviating IBD is mainly that the active ingredients in HC and related preparations can regulate the intestinal microenvironment, including regulating intestinal flora and its metabolites, thereby improving the body’s immunity to alleviate IBD (as shown in **Figure 5**). This effect has been verified not only in animal experiments, but also in clinical human drug evaluation.

However, due to the presence of aristolochic acid lactam alkaloids (83), which have been confirmed to be nephrotoxic and carcinogenic, in HC, the development, research, and utilization of HC in IBD have been limited, and there is a lack of relevant literature reports. However, current research presents divergent opinions, suggesting that the nephrotoxic and carcinogenic nature of Aristolochic acid alkaloids primarily pertains to Aristolochic acid I, originating from plants within the Aristolochiaceae family such as Manshurian Dutchmanspipe Stem and Dutchmanspipe Vine. In contrast, HC belongs to the Saururaceae family, and its contained alkaloids are of the Aristolochic acid II type, including BII, AII, and FII (84–86). Their content in the total alkaloids of HC is only 0.016 g/kg, thus posing minimal risk of kidney damage and carcinogenicity (87). A cross-sectional study conducted in China to investigate the dietary habits of HC among various regional populations has affirmed this observation. Among the 3,561 volunteers included in the study, no significant correlation was found between the consumption of HC and kidney diseases or malignancies of the urinary system (R=0.988). Even after controlling for confounding factors such as age and region, the results remained negative (88). Similar animal toxicology experiments have yielded consistent findings (89).

Therefore, we should further develop the research and development of active ingredients and preparations related to HC to alleviate IBD. It is not only rich in resources and easy to obtain, but also contains many bioactive substances. In addition to the above-mentioned components, there are also chlorogenic acid, neochlorogenic acid, cryptochlorogenic acid, caffeic acid, succinic acid, β-sitosterol-4-en-3-one, β-sitosterol-3,6-dione, and daucosterol, which have great potential for development and utilization (90).

## 4 Summary and prospect

HC, a naturally occurring medicinal plant with dual therapeutic and dietary properties, has been substantiated through research as a beneficial supplement for the treatment of IBD. In this article, we discuss the potential mechanism of HC in the treatment of IBD based on its main active ingredients and related preparations, and emphasize its role in alleviating IBD by regulating the intestinal microenvironment and improving the body’s immunity. Although IBD is a complex disease, combined with literature reports, the active ingredients in HC and related traditional Chinese medicine preparations may regulate the intestinal microenvironment to alleviate IBD, providing a promising treatment approach for patients to alleviate the symptoms of IBD.

However, it is essential to acknowledge that current research is still in its preliminary stages and presents several unresolved mysteries and challenges. To gain a better understanding of the mechanism of action of HC, further investigations are required, including laboratory researches, clinical trials, identification of bioactive components, and optimization studies of treatment dosages and durations. Furthermore, comprehensive assessments of drug interactions and safety are also necessary to better serve patients, enhance their quality of life, and offer more avenues and options for the treatment of IBD.

## Author contributions

SW: Conceptualization, Writing – original draft, Writing – review & editing. LL: Validation, Writing – original draft, Writing – review & editing. YC: Visualization, Writing – review & editing. QL: Data curation, Writing – review & editing. SZ: Visualization, Writing – review & editing. NL: Methodology, Visualization, Writing – review & editing. YW: Supervision, Writing – review & editing. JY: Funding acquisition, Supervision, Writing – review & editing.
